# Effect of Different *Agrobacterium rhizogenes* Strains on Hairy Root Induction and Phenylpropanoid Biosynthesis in Tartary Buckwheat (*Fagopyrum tataricum* Gaertn)

**DOI:** 10.3389/fmicb.2016.00318

**Published:** 2016-03-14

**Authors:** Aye Thwe, Mariadhas Valan Arasu, Xiaohua Li, Chang Ha Park, Sun Ju Kim, Naif Abdullah Al-Dhabi, Sang Un Park

**Affiliations:** ^1^Department of Crop Science, Chungnam National UniversityDaejeon, South Korea; ^2^Department of Botany and Microbiology, Addiriyah Chair for Environmental Studies, College of Science, King Saud UniversityRiyadh, Saudi Arabia; ^3^Department of Bio-Environmental Chemistry, Chungnam National UniversityDaejeon, South Korea

**Keywords:** tartary buckwheat, *Agrobacterium rhizogenes*, polypropanoid biosynthetic genes, rutin, anthocyanin, HPLC

## Abstract

The development of an efficient protocol for successful hairy root induction by *Agrobacterium rhizogenes* is the key step toward an *in vitro* culturing method for the mass production of secondary metabolites. The selection of an effective *Agrobacterium* strain for the production of hairy roots is highly plant species dependent and must be determined empirically. Therefore, our goal was to investigate the transformation efficiency of different *A. rhizogenes* strains for the induction of transgenic hairy roots in *Fagopyrum tataricum* ‘Hokkai T10’ cultivar; to determine the expression levels of the polypropanoid biosynthetic pathway genes, such as *ftpAL, FtC4H, Ft4CL, FrCHS, FrCH1, FrF3H, FtFLS1, FtFLS2, FtF3^,^ H1, FtF3′H2, FtANS*, and *FtDFR*; and to quantify the *in vitro* synthesis of phenolic compounds and anthocyanins. Among different strains, R1000 was the most promising candidate for hairy root stimulation because it induced the highest growth rate, root number, root length, transformation efficiency, and total anthocyanin and rutin content. The R1000, 15834, and A4 strains provided higher transcript levels for most metabolic pathway genes for the synthesis of rutin (22.31, 15.48, and 13.04 μg/mg DW, respectively), cyanidin 3-*O*-glucoside (800, 750, and 650 μg/g DW, respectively), and cyanidin 3-*O*-rutinoside (2410, 1530, and 1170 μg/g DW, respectively). A suitable *A. rhizogenes* strain could play a vital role in the fast growth of the bulk amount of hairy roots and secondary metabolites. Overall, R1000 was the most promising strain for hairy root induction in buckwheat.

## Introduction

*Fagopyrum tataricum* (Gaertn) is commonly known as tartary buckwheat. It belongs to the Polygonaceae family and is mainly cultivated in the mountain regions of China, Korea, Japan, India, USA, Europe, Brazil, Canada, Australia, and South Africa. *F. tataricum* is known as a functional food (due to the presence of proteins and amino acids) and as a traditional medicinal plant (due to the presence of phenolic compounds). Tartary buckwheat has a bitter taste. Therefore, its consumption has decreased. However, the plant has received increased attention due to the presence of significant amounts of pharmacologically important phenolic compounds, such as quercetin-3-glycoside, kaempferol-3-glycoside, chlorogenic acid, iso-orientin, orientin, rutin, vitexin, and quercitrin (Lee et al., 2014). In addition, it contains dietary fibers, proteins, starch, polyunsaturated fatty acids, and vitamin B and C complexes (Bonafaccia et al., 2003). Phenolic compounds, such as rutin and anthocyanins, that have been identified in buckwheat showed better *in vitro* antioxidant activity and *in vitro* pharmacological functions, such as cholesterol reduction, tumor inhibition, anti-hypertension, and control of diabetes and carcinogenesis (Kayashita et al., 1997; Chan, 2003; Kawa et al., 2003; Ishii et al., 2008).

Various strategies have been demonstrated for the synthesis and production of pharmaceutically important phytochemicals (Nagella et al., 2013). These strategies include *Agrobacterium*-mediated gene transfer, *in vitro* cell line establishment, *in vitro* cell suspension cultures, bioreactor cultivation, shoot cultivation, organ cultivation at the bioreactor level, and *in vitro* hairy root cultivation (Bourgaud et al., 2001). However, among these techniques, *Agrobacterium*-mediated gene transfer has been widely studied as a strategy for producing hairy root lines with high yield for plant compounds (Ali et al., 2008). *Agrobacterium* is a Gram-negative soil-borne bacterium that is able to transfer part of its DNA (i.e., T-DNA carried on a large plasmid) to a host plant cell (Tzfira et al., 2004). The hairy roots obtained by infecting plants with *Agrobacterium rhizogenes* are unique with respect to their genetic and biosynthetic stability and have been intensively used to induce a stable and high yield production of selected plant metabolites. *A. rhizogenes* are appropriate for the production of valuable secondary metabolites, because they can enhance growth regulators and are characterized by fast growth (Shen et al., 1988; Wysokinska and Chmiel, 1997; Giri and Narasu, 2000). Currently, modern molecular techniques are used to regulate metabolic pathway genes that are required for the production of secondary metabolites (Georgiev et al., 2012; Sharma et al., 2013). Additionally, hairy roots are considered as a potential source of new natural compounds. Li et al. (1998) obtained a novel antimicrobial compound, licoagrodione, from *Glycyrrhiza glabra* using a hairy root culture, and a new tropane alkaloid ester was obtained from *Datura stramonium* using a hairy root culture (Berkov et al., 2003). Furthermore, growth regulators are not required for the hairy root cultivation, which is an important consideration because some plant hormones are toxic (e.g., 2,4-dichlorophenoxyacetic acid; Shanks and Morgan, 1999). Therefore, hairy root culture provides possibilities for isolating and synthesizing new compounds with high pharmaceutical properties (Kwon et al., 1997).

Many strains of *A. rhizogenes* exist and have been used for plant transformation. Recently, Ooi et al. (2013) and Sudha et al. (2013) used *A. rhizogenes* to regulate genes that were involved in the plant secondary metabolite production. The selection of an effective *Agrobacterium* strain for the production of transformed root cultures significantly depends on the plant species and must be determined empirically. The differences in virulence, morphology, and growth rate are at least partially related to the variety of Ri (root inducing)-plasmids within each bacterial strain (Park and Facchini, 2000). Among the T-DNA genes in Ri-plasmid, the rol oncogenes cause striking phenotypical and biochemical alterations in the transformed hairy root. The *rol* genes are potential activators of the secondary metabolism in transformed cells from the Solanaceae, Araliaceae, Rubiaceae, Vitaceae, and Rosaceae families (Bulgakov et al., 2008). It is largely unknown whether different *A. rhizogenes* strains vary in their abilities to stimulate secondary metabolite production.

**FIGURE 1:**
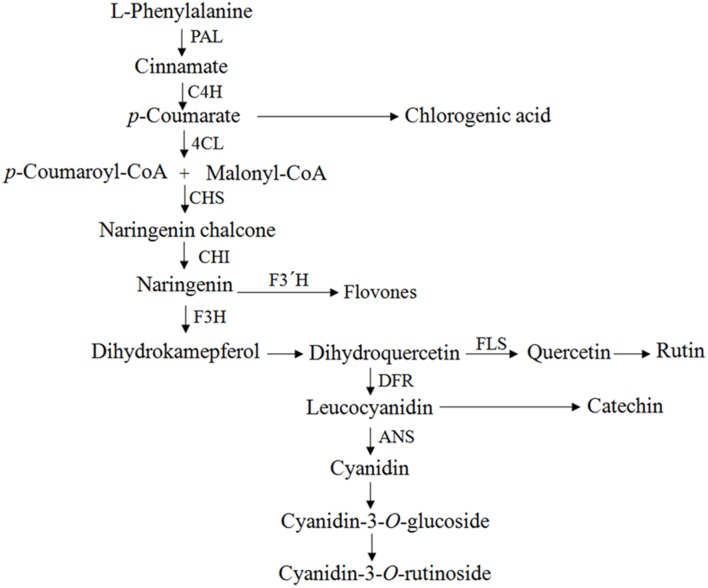
**Flavonoid pathway scheme.** PAL, phenylalanine ammonia lyase; C4H, cinnamate 4-hydroxylase; 4CL, 4-coumaroyl CoA ligase; CHS, chalcone synthase; CHI, chalcone isomerase; F3H, flavones 3-hydroxylase; FLS, flavonol synthase; F3’H, flavonoid 3′-hydroxylase; ANS, anthocyanidin synthase; DFR, dihydroflavonol-4 reductase.

In our previous study, we claimed that *F. tataricum* infected with *A. rhizogenes* produced significant amounts of phenylpropanoid (Thwe et al., 2013) (**Figure 1**). However, to date, there are no reports on the transformation of *F. tataricum* via different *A. rhizogenes* strains for the phenylpropanoid production. Therefore, in the present study, we aimed to develop a novel hairy root culture method for the production of pharmacologically important phenolic compounds. Different *A. rhizogenes* strains were screened for the infection, and the expression levels of phenylpropanoid biosynthetic genes were evaluated.

## Materials and Methods

### Chemicals and Reagents

HPLC-grade methanol, acetonitrile, and formic acid were obtained from Wako Pure Chemical Industries (Osaka, Japan). Phenolic compound standards for rutin, catechin, chlorogenic acid, benzoic acid, ferulic acid, and quercetin were procured from Extrasynthese (Genay, France). Cyanidin 3-*O*-rutinoside and cyanidin-3-*O*-glucoside were purchased from Fujicco Co., Ltd. (Kobe, Japan). Other routine reagents and chemicals were procured from Sigma–Aldrich (St. Louis, MO, USA).

### Plant Materials

Tartary buckwheat (‘Hokkai T 10’) seeds were kindly gifted by the Hokkaido Agricultural Research Center, Sapporo, Japan, in July 2011. For the hairy root stimulation, the seeds were disinfected with 70% (v/v) ethanol for 120 s, followed by rinsing with 4% (v/v) sodium hypochlorite solution (NaClO) for 15–20 min and with Tween-20 for 15 min. The seeds were mixed with ice-cold autoclaved sterile distilled water three to five times and completely washed five times to remove any trace elements. The surface-sterilized seeds were wiped with sterile cotton to remove water droplets and, then the seeds were transferred into a sterile culture flask containing 25 ml of autoclaved half-strength MS (Murashige and Skoog, 1962) without hormones. The seeds were incubated at 25°C under light with an intensity of 35 μmol s^-1^ m^-2^ for 3 days to facilitate seed germination. The sterile bottle with seeds was maintained in a Magenta box containing 50 ml of the same basal medium under 16/8-h light/dark conditions for the growth of seedlings at 25 ± 1°C.

### Bacterial Strains and Maintenance

Wild-type *A. rhizogenes* (R1000), R1200, R13333, R15834, R1601, LBA9402, and A4 were used to determine the transformation efficiency. The strains were kindly gifted by Centro de Investigacion Cientifica de Yucatan, Mexico, and were maintained in our laboratory. The bacterial strains were cultivated in an LB medium containing 1 g of tryptone, 0.5 g of yeast extract, and 0.5 g of NaCl in a 100-ml flask at 28°C and 224 rcf for 24 h. All of the strains were maintained in 20% glycerol at –80°C. Viability of the strains was determined intermittently by growing in LB agar.

### Hairy Root Induction

Hairy root induction was performed using a slightly modified method of Thwe et al. (2013). Briefly, 5 ml of freshly cultivated *A. rhizogenes* strains at the mid-log phase (A_600_ = 0.6) were centrifuged at 5600 rcf for 10 min. Cell pellets were further washed with an ice-cold phosphate buffer (pH 7) and re-suspended in half-strength MS liquid medium. The explants were dipped into suspensions of different *A. rhizogenes* strains for 20 min, dried on a sterile tissue paper in a sterile bottle, and incubated under dark conditions at 25 ± 1°C on a half-strength solid MS medium for 2 days. After incubation, the explants were carefully collected and rinsed with sterile distilled water and cultivated in a hormone-free half-strength MS medium with 500 mg/l of cefotaxime. After 7 days of incubation, the emerged hairy roots were further sub-cultured in a flask containing a half-strength MS medium for 21 days at 25 ± 1°C. After incubation, the hairy roots were harvested to examine the gene expression levels and to quantify the phenolic compounds. The collected hairy roots were carefully stored in a clean container at –80°C for further chemical and molecular analyses.

### Extraction of Genomic DNA and PCR Analysis

Genomic DNA of transformed tartary buckwheat was extracted using a DNeasy Plant Mini Kit (Qiagen; Valencia, CA, USA). The primers for the fragments of *rol* genes (*rol* A, B, C, and D) are listed in **Table 1**. The conditions for thermal amplification of the *rol* genes were as follows: initial denaturation at 95°C for 2 min, followed by 30 cycles of amplification at 95°C for 30 s, primer annealing at 55°C for 45 s, and primer extension at 72°C for 1 min and final extension at 10 min and cooling to 4°C. The purity of amplified products was checked by mixing 10 μl of the PCR products with a loading dye and by performing electrophoresis on 1% agarose gels that were prepared in a 0.5*TBE (Tris/Borate/EDTA) buffer. The gels were analyzed for fragment sizes of the *rol* genes (*rol* A, B, C, and D).

**Table 1:** Primer information for PCR analysis.

Primer	Sequence (5′ to 3′)
*rol* A-Forward	CATGTTTCAGAATGGAATTA
*rol* A-Reverse	AGCCACGTGCGTATTAATCC
*rol* B-Forward	TCACAATGGATCCCAAATTG
*rol* B-Reverse	TTCAAGTCGGCTTTAGGCTT
*rol* C-Forward	ATGGCTGAAGACGACCTGTGT
*rol* C-Reverse	TTAGCCGATTGCAAACTTGCA
*rol* D-Forward	ATGGCCAAACAACTTTGCGA
*rol* D-Reverse	TTAATGCCCGTGTTCCATCG

### Extraction of the Total RNA and cDNA Synthesis

The RNeasy Plant Mini Kit (Qiagen; Valencia, CA, USA) was used to extract the total RNA from the wild and transformed tartary buckwheat hairy roots. The purity of RNA was evaluated using 1.2% gels that were stained with ethidium bromide and by measuring absorbance at 260:280 nm wavelength using a NanoVue Plus spectrophotometer. In total, 1 mg of DNA-free total RNA was used to synthesize cDNA using transcriptase. The synthesized cDNA was further used for real time (RT)-PCR.

### Expression Patterns of Phenylpropanoid Biosynthetic Cluster Genes

The BIO-RAD CFX96 Real-time PCR system (Bio-Rad Laboratories, Hercules, CA, USA) was used to determine the transcriptional levels of phenylpropanoid biosynthetic genes from the wild and transformed tartary buckwheat hairy roots. The primers for phenylpropanoid biosynthetic genes were designed as described by Li et al. (2010). Quantitative RT-PCR analysis was performed using gene-specific primer sets under the following conditions: initial denaturation at 95°C for 3 min, followed by 40 cycles at 95°C for 15 s, annealing at 55°C for 30 s, and elongation at 72°C for 20 s. The transcript levels of mRNA were compared relative to those of the standard histone H3 gene. The variations in expression levels were calculated by comparing three replicates of each sample.

### Extraction and Quantification of Phenolic Compounds Using HPLC

To quantify the phenolic compounds in the transformed tartary buckwheat hairy roots, 100 mg of the freeze-dried hairy root samples was powdered using a mortar and a pestle and was rinsed vigorously with 3 ml of 100% methanol and, then, heated at 60°C for 1 h in a sonicator. The slurry was mixed intermittently every 20 min during the extraction. After 1 h, the samples were centrifuged at 21,952 rcf for 10 min, and the supernatant was filtered through a 0.45-mm PTFE syringe filter for the HPLC analysis with a Futecs model NS-4000 HPLC apparatus (Daejeon, Korea). The operating conditions for HPLC and the separating protocols for individual phenolic compounds were according to the method of Li et al. (2010). Briefly, 20 μl of the samples were injected; the HPLC column temperature was maintained at 30°C; individual compounds were detected at 280 nm. Mobile solutions were passed through the column at 1 ml/min. The solutions consisted of a mixture of (A) MeOH:water:acetic acid (5:92.5:2.5, v/v/v) and (B) MeOH:water:acetic acid (95:2.5:2.5, v/v/v). The initial mobile phase composition was 0% solvent B, followed by a linear gradient from 0 to 80% solvent B over 48 min, and holding at 0% solvent B for an additional 10 min. All phenolic compounds were calculated by comparing the HPLC peak areas with those of authentic standards, according to the procedures of Li et al. (2010).

### Quantification of Individual Anthocyanin Content Using HPLC

For extraction of anthocyanins, 100 mg of the powdered freeze-dried samples was mixed with 2 ml of water:formic acid 95:5 (v/v) and maintained in the dark under shaking conditions. After proper mixing for 5 min, the samples were incubated in a sonicator for 20 min with intermittent vortexing. After thorough shaking, the mixture was centrifuged at 11,200 rcf for 15 min at 4°C. The collected debris-free supernatant was filtered and stored in a sterile brown bottle for HPLC analysis. Perkin Elmer Flexar HPLC equipped with a Security Guard Cartridges Kit AQ C18 column (4 mm; 63 mm, i.d.) and a Synergi 4 m POLAR-RP 80A column (250 mm; 64.6 mm, i.d., 4 mm, particle size; Phenomenex, Torrance, CA, USA) was used for the separation and quantification of the individual anthocyanin content. The sample was eluted with a mixture of (A) water:formic acid (95:5, v/v) and (B) acetonitrile:formic acid (95:5, v/v). The following gradient program was used: 0–8 min, 5–10% solvent B; 8–13 min, 10–13% solvent B; 13–15 min, 13% solvent B; 15–18 min, 13–15% solvent B; 18–25 min, 15% solvent B; 5% solvent B at 25.1 min; and, finally, 5% solvent B for 10 min (total, 35 min). Cyanidin-3-*O*-glucoside and cyanidin-3-*O*-rutinoside standards were used for the quantification of individual anthocyanin content in the samples.

### Statistical Analysis

A statistical analysis system (SAS version 9.2) was used for comparative analysis of gene expression of individual genes and phenolic components in the wild and transformed hairy roots of tartary buckwheat.

## Results

### Establishment of the Hairy Root Culture

**FIGURE 2:**
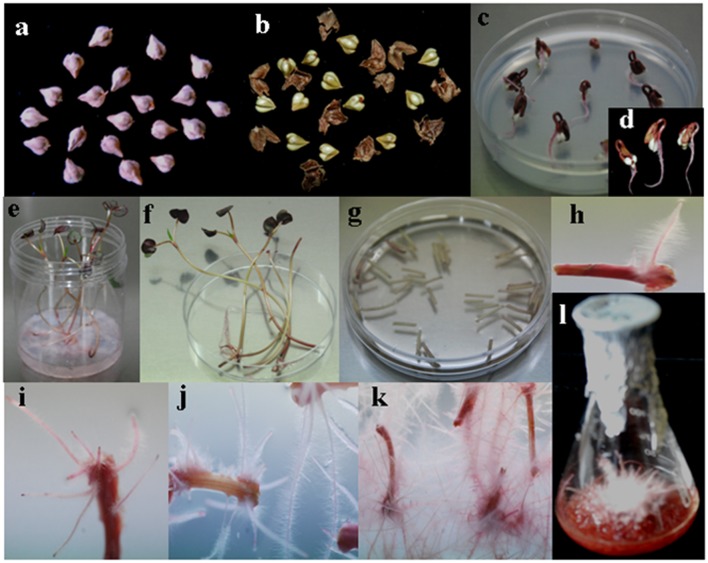
**Sequential stages of hairy root induction in *Fagopyrum tataricum* ‘Hokkai T10’. (a)** Hokkai T10 seeds. **(b)** De-hulled seeds. **(c)** Germinated seeds on a ½ MS solid medium. **(d)** Three-day-old seedlings. **(e)** Five-day-old seedlings transferred to the Magenta box. **(f)** Explant preparation for *Agrobacterium* infection. **(g)** Explant infection in *Agrobacterium* inoculum. **(h)** Hairy root induction. **(i)** Profuse hairy roots 10 days after infection. **(j)** Hairy root growth 2 weeks after infection. **(k)** Rapid hairy root growth on a fresh medium. **(l)** Hairy root growth 14 days after culture in a ½ MS liquid medium.

**FIGURE 3:**
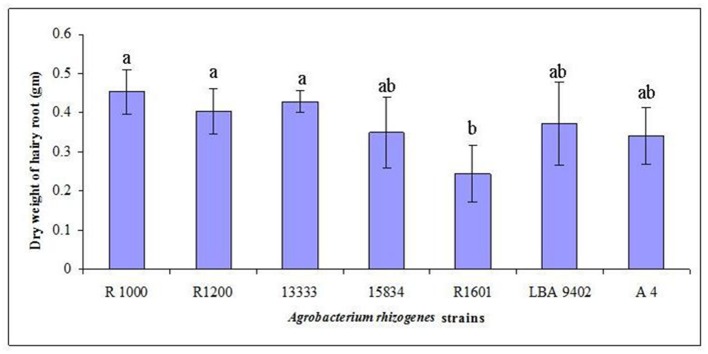
**Development of *F. tataricum* ‘Hokkai T10’ hairy roots as influenced by different *A. rhizogenes* strains.** Data from three replicates were compared 14 days after culturing in a ½ MS liquid medium. Mean values indicated by the same letter in a column are not different at *P* < 0.05, according to Duncan’s multiple range test.

Wild-type *A. rhizogenes* (R1000) and strains R1200, R13333, R15834, R1601, LBA9402, and A4, that were isolated from crown gall disease plants, were used to determine the transformation efficiency. Mature seeds of tartary buckwheat were treated with different strains of *A. rhizogenes* to develop the hairy root culture. Primary results confirmed that the induction of hairy roots in tartary buckwheat is strain-specific because different *A. rhizogenes* strains exhibited varied infection efficiency patterns, hairy roots formation rates, and hairy root lengths. Among the *A. rhizogenes* strains R1000, R1200, LBA 9402, and A4, a 100% infection efficiency was observed. Whereas, the strain 15834 had comparatively lower efficiency (65%; **Table 2**). Interestingly, each strain showed a similar infection type related to hairy roots and hairy root length. With respect to the hairy root length and the number of hairy roots, the strain R1000 (average, 2.93 cm and 5.3) was superior, followed by A4 and LBA 9402. Other strains showed a moderate number of hairy roots and slightly shorter hairy roots. However, no significant variations were observed among R1000, R1200, and 13333 strains. The occurrence of necrotic explant tissues in the form of hairy roots on a MS medium was fast and clearly indicated the transformation efficiency. Furthermore, the hairy root induction was comparatively faster (**Figure 2**). The dry weight of the developed hairy roots indicated that R1000 was dominant (0.4 g), followed by R3333 (0.41 g). Whereas, R1601 exhibited the lowest dry weight (**Figure 3**). Incubation period indicated the accumulation of phenolic compounds. PCR amplification of the related *rol* A, B, C, and D genes (300 bp, 750 bp, 520 bp, and 1000 bp, respectively) in all of the transformed tartary buckwheat hairy roots confirmed the successful transformation.

**Table 2:** Effects of different *Agrobacterium rhizogenes* strains on hairy root growth of *Fagopyrum tataricum* ‘Hokkai T10’.

*Agrobacterium* strain	Infection efficiency (%)	Number of hairy roots/explants	Length of hairy root (cm)
R 1000	100	5.3 ± 0.6a	2.93 ± 0.12a
R1200	100	3.1 ± 0.32c	2.27 ± 0.23b
13333	70	2.5 ± 0.06d	2.2 ± 0.35b
15834	60	1.9 ± 0.32e	1.53 ± 0.06c
R1601	65	2.1 ± 0.06e	2.53 ± 0.12b
LBA 9402	100	3.8 ± 0.06bc	2.33 ± 0.15b
A4	100	3.9 ± 0.12b	2.43 ± 0.06b

### Expression of Phenylpropanoid Biosynthetic Pathway Genes

**FIGURE 4:**
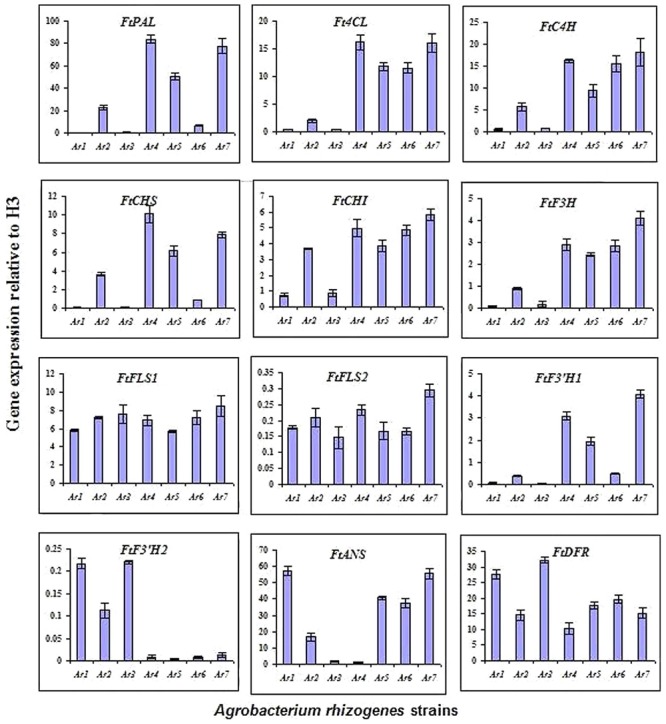
**Expression levels of phenylpropanoid biosynthetic pathway genes observed using qRT-PCR at 14 days after culturing of hairy roots.** Expression level of each gene is relative to that of H3 (*Ar1* = R1000, *Ar2* = R1200, *Ar3* = 13333, *Ar4* = 15834, *Ar5* = R1601, Ar6 = LBA9402, *Ar7* = A4).

To evaluate the expression levels of the genes related to the phenylpropanoid biosynthesis pathway in tartary buckwheat, the following genes were studied: *ftpAL, FtC4H, Ft4CL, FrCHS, FrCH1, FrF3H, FtFLS1, FtFLS2, FtF3′H1, FtF3′H2, FtANS*, and *FtDFR.* These genes encode PAL (phenylalanine ammonia-lyase), C4H (cinnamate 4-hydroxylase), 4CL (4-coumaroyl-CoA ligase), CHS (chalcone synthase), CHI (chalcone isomerase), F3H (flavone 3-hydroxylase), FLS (flavonol synthase), F3′H (flavonoid 3′-hydroxylase), ANS (anthocyanidin synthase), and DFR (dihydroflavonol-4 reductase), respectively. Expression patterns of the selected genes in the transformed roots were strain-specific. All nine strains exhibited a wide range of gene expressions, which clearly indicated that each strain had a different gene regulation pattern in the tartary buckwheat host. In most cases, the transcript levels were comparatively higher for the strains 15834, A4, and R1000 (**Figure 4**). In general, the expression patterns of *FtFLS1* and *FtFLS2* were similar in all *A. rhizogenes* strains. R15834 showed a higher expression pattern compared with other strains. In contrast, R1000 did not enhance the expression levels of regulatory genes, such as *ftpAL, FtC4H, Ft4CL, FtCHS*, *FtCHI, FtF3H*, and *FtF3′H-1*. In roots that were infected with R1000, R1200, and 13333, the expression levels of *FtFLS1, FtFLS2, FtF3′H2*, and *FtDFR* were significantly higher relative to the transcript levels of the housekeeping gene (histone H3). These genes are directly involved in the biosynthesis of phenolic compounds in buckwheat. However, their relative expression in strains 15834, R1601, LBA9402, and A4 was lower compared with the other strains. In addition, LBA9402 and A4 showed significant expression for key enzymes related to the anthocyanin biosynthetic pathway (*FtANS*).

### Quantification of Phenolic Compounds in Hairy Roots of Tartary Buckwheat

Individual content of phenolic compounds in hairy roots of tartary buckwheat is listed in **Table 3**. Six phenolic compounds were detected using HPLC. Irrespective of the strain, all six phenolic compounds were observed in the transformed hairy roots. Among the phenolic compounds, rutin (7.14–23.31 μg/mg dry weight) showed the dominant amount in tartary buckwheat, whereas the content of rutin in the roots infected with R1000, R1200, 13333, and 15834 contributed an average of 92% of the total phenolic compounds. Infection with R1000 produced the highest rutin content (22.31 μg/mg dry weight). The observed levels of catechin, chlorogenic acid, ferulic acid, benzoic acid, and quercetin were less than 1 μg/mg dry weight.

**Table 3:** Content of phenolic compounds in hairy roots induced by different *Agrobacterium rhizogenes* strains (μg/mg dry weight).

*Agrobacterium* strain	Catechin	Chlorogenic acid	Ferulic acid	Benzoic acid	Rutin	Quercetin
R1000	0.53 ± 0.02a	0.50 ± 0.01a	0.30 ± 0.01c	0.16 ± 0.02a	22.31 ± 0.54a	0.34 ± 0.03a
R1200	0.53 ± 0.01a	0.45 ± 0.01b	0.27 ± 0.01c	0.14 ± 0.04a	21.96 ± 0.65a	0.35 ± 0.01a
13333	0.36 ± 0.06b	0.40 ± 0.09bc	0.42 ± 0.01b	0.10 ± 0.01b	18.58 ± 0.16b	0.34 ± 0.01a
15834	0.20 ± 0.01c	0.34 ± 0.02c	0.45 ± 0.01b	0.09 ± 0.01b	15.48 ± 0.56c	0.30 ± 0.02a
R1601	0.19 ± 0.01c	0.43 ± 0.03b	0.43 ± 0.04b	0.07 ± 0.01b	7.14 ± 0.68e	0.39 ± 0.01a
LBA 9402	0.19 ± 0.01c	0.43 ± 0.03b	0.43 ± 0.04b	0.07 ± 0.01b	7.14 ± 0.68e	0.39 ± 0.01a
A4	0.17 ± 0.01c	0.47 ± 0.01ab	0.76 ± 0.01a	0.07 ± 0.01b	13.04 ± 0.01cd	0.32 ± 0.02a

### Quantification of Anthocyanins in Hairy Roots of Tartary Buckwheat

Cyanidin derivatives of anthocyanins, such as cyanidin 3-*O*-glucoside and cyanidin 3-*O*-rutinoside, were detected in the hairy roots of tartary buckwheat using HPLC (**Table 4**). The results indicated that the amount of cyanidin 3-*O*-rutinoside (610–2400 μg/g dry weight) was higher than that of cyanidin 3-*O*-glucoside (300–800 μg/g dry weight) in the infected tartary buckwheat roots. R1000-infected roots produced higher amounts of anthocyanins (3190 μg/g dry weight), followed by R1200- (2490), 13333- (2330), and 15834-infected roots (2280), whereas LBA 9402-infected roots showed the least total anthocyanin content (910). The average levels of cyanidin 3-*O*-glucoside and cyanidin 3-*O*-rutinoside in the treated samples were 400 and 1500 μg/g dry weight, respectively.

**Table 4:** Content of anthocyanin in hairy roots induced by different *Agrobacterium rhizogenes* strains (μg/g dry weight).

*Agrobacterium* strain	Cyanidin 3-*O*-glucoside	Cyanidin 3-*O*-rutinoside	Total
R 1000	800 ± 0.00a	2400 ± 20.0a	3190 ± 20.0a
R1200	570 ± 10.0d	1910 ± 60.0b	2490 ± 60.0b
13333	490 ± 0.00e	1840 ± 40.0c	2330 ± 40.0c
15834	750 ± 10.0b	1530 ± 30.0d	2280 ± 30.0c
R1601	490 ± 10.0e	1080 ± 20.0f	1570 ± 20.0e
LBA 9402	300 ± 10.0f	610 ± 10.0g	910 ± 20.0f
A 4	650 ± 10.0c	1170 ± 10.0e	1820 ± 20.0d

## Discussion

Plant metabolites and functional compounds have attracted interest because of their desired pharmaceutical properties and various useful applications in the medical field (Banerjee et al., 2012; Kaliyan and Agastian, 2015). Among plants, buckwheat is considered as a stable healthy food worldwide because of the presence of a wide variety of phytochemicals and nutrients, such as flavonoids, phenolic compounds, amino acids, and vitamins (Golisz et al., 2007; Alvarez-Jubete et al., 2010; Sedej et al., 2010). Buckwheat is a rich source of rutin (Hagels et al., 1995; Lachman et al., 2000). Rutin accumulates to the highest extent in buckwheat and cannot be found in other grains, such as wheat, rice, and corn. Thus, buckwheat is considered to be a major dietary source of rutin. Rutin has a wide range of pharmacological properties, such as anti-allergic, antioxidant, antimicrobial, antidiabetic, and anticancer properties (Al-Dhabi et al., 2015). Therefore, an accurate and reliable transformation method for the increased production of phenolic compounds, such as rutin and other phytochemicals in tartary buckwheat, is desired. *A. tumefaciens* and *A. rhizogenes* play an important role in the transformation of plant cell culture for the improved production of novel compounds. The development of hairy root culture using *A. rhizogenes* has various advantages over callus and cell culture due to gene stability and a relatively low cost and cultivation requirements (Banerjee et al., 2012). Moreover, *A. rhizogenes*, with its higher infection efficiency toward the plants, established new trends in the field of functional research for the production of major molecules that are similar to phenolic compounds. In the present study, the transformation efficiency of different *A. rhizogenes* strains, expression pattern of biosynthetic genes related to the production of phenolic compounds, and production capability of phenolic compounds were determined by inducing hairy roots in tartary buckwheat. The results indicated that all of the tested *A. rhizogenes* strains were able to induce hairy root formation with different transformation efficiencies. Zehra et al. (1999) studied the transformation efficiency of different *A. rhizogenes* strains for the production of novel metabolites in plants, and they confirmed that the hairy root induction is strain-specific. Generally, the A_4_GUS strain was used for the hairy root induction in medicinal plants, such as *Scutellaria baicalensis*, *Gentiana macrophylla*, *Hypericum perforatum*, *Aesculus hippocastanum*, and *Catharanthus roseus* (Batra et al., 2004; Zdravković-Korać et al., 2004; Vinterhalter et al., 2006; Tiwari et al., 2007, 2008). Previously, we confirmed that *rol* gene insertions were observed in all of the strains and that the *rol* genes of the Ri plasmid in *A. rhizogenes* are responsible for the induction of hairy roots. However, the transformation efficiency of R1000 was superior to that of the other tested strains. Similarly, Tao and Li (2006) and Tiwari et al. (2007) reported that R1000 was efficient for developing a hairy root culture for medicinal plants.

Previously, we established a hairy root culture of *F. tataricum* and *F. esculentum* to produce phenolic compounds (Kim et al., 2009, 2010). To determine the gene transfer efficiency of different *A. rhizogenes* strains in the phenylpropanoid biosynthesis pathway in buckwheat, the expression levels of polypropanoid regulatory genes were studied. The results confirmed that, in most cases, the expression levels were comparatively higher for the R1000, A4, and 15834 strains. In addition, the expression levels for *FtANS* genes were directly related to anthocyanin biosynthesis in the transformed hairy roots. Similarly, Park et al. (2011) and Thwe et al. (2013) reported increased expression levels of *FtANS* genes in Hokkai T10’ relative to in ‘Hokkai T8’ roots by R1000-mediated transformation. However, recently, Seo et al. (2015) claimed that the expression levels of FtDFR and FtFLS2 were similar. The increased transcript levels of FtDFR and FtANS were directly related to the content of leucoanthocyanidins and anthocyaninidins, which are precursors for the biosynthesis of cyanidin 3-*O*-glucoside and cyanidin 3-*O*-rutinoside, respectively. Our study confirmed that the expression levels of the regulatory genes for polypropanoid biosynthesis in buckwheat are directly related to the gene transfer efficiency of *A. rhizogenes* strains. Accumulation of the anthocyanin and phenolic compounds in hairy roots from the infection of different *A. rhizogenes* strains was not consistent with the expression of phenylpropanoid biosynthetic genes. We assumed that the expression level of phenylpropanoid biosynthetic genes in hairy roots from the infection of different *A. rhizogenes* strains would be different with respect to the source of the *A. rhizogenes* strains. However, we determined the expression of phenylpropanoid biosynthetic genes when we harvested the hairy roots.

Analysis of the individual phenolic compounds and anthocyanins contents in the transformed hairy root cultures showed the expression levels of the target genes in polypropanoid biosynthesis. Among the phenolic compounds, rutin and chlorogenic acid showed dominant levels in the hairy root culture. Previous studies have reported that the dry weight of tartary buckwheat seeds was 1.7% (Seo et al., 2015). Because rutin, which is present in tartary buckwheat, has various biological applications, its content can be increased by the hairy root culture. Similarly, the levels of anthocyanins, which have antioxidant, anti-inflammatory, antiviral, and antihistamine properties, could be enhanced in tartary buckwheat (Rice-Evans and Packer, 2003). Trotin et al. (1993) and Lee et al. (2007) developed buckwheat hairy root cultures to enhance the production of phenolic compounds, such as rutin, chlorogenic acid, and caffeic acid. Overall, R1000 has been recommended as a promising strain for the hairy root induction and for the phenolic compound accumulation in buckwheat.

## Conclusion

Gene transfer and hairy root stimulation in tartary buckwheat were affected by the *A. rhizogenes* strains used. Specifically, the genes that were involved in the metabolic pathway for the biosynthesis of polypropanoid were studied using nine *A. rhizogenes* strains. Transcript analysis clearly indicated that the expression of individual genes was strain-specific. However, mass production of hairy roots in buckwheat was efficiently established using the *A. rhizogenes* R1000 strain by following the protocol described in this study. Overall, the selection and identification of a suitable *A. rhizogenes* strain is useful for improving the production of phenolic compounds and anthocyanins in buckwheat.

## Author Contributions

All authors listed, have made substantial, direct and intellectual contribution to the work, and approved it for publication.

## Conflict of Interest Statement

The authors declare that the research was conducted in the absence of any commercial or financial relationships that could be construed as a potential conflict of interest.
